# Development of a platform for quantum-dot-labeled polyethylene nanoplastics for dynamic analytical applications

**DOI:** 10.1039/d6ra01781a

**Published:** 2026-04-07

**Authors:** Mii Hokaku, Yuya Haga, Kosuke Tanaka, Hirofumi Tsujino, Haruyasu Asahara, Kazuma Higashisaka, Yasuo Tsutsumi

**Affiliations:** a Graduate School of Pharmaceutical Sciences, The University of Osaka 1-6 Yamadaoka, Suita Osaka 565-0871 Japan haga-y@phs.osaka-u.ac.jp ytsutsumi@phs.osaka-u.ac.jp; b School of Pharmaceutical Sciences, The University of Osaka 1-6 Yamadaoka, Suita Osaka 565-0871 Japan; c Material Cycles Division, National Institute for Environmental Studies Tsukuba Ibaraki 305-8506 Japan; d Museum Links, The University of Osaka 1-13 Machikaneyamacho Toyonaka Osaka 560-0043 Japan; e Institute for Open and Transdisciplinary Research Initiatives, The University of Osaka 1-1 Yamadaoka, Suita Osaka 565-0871 Japan; f Institute for Advanced Co-Creation Studies, The University of Osaka 1-6 Yamadaoka, Suita Osaka 565-0871 Japan; g Graduate School of Medicine, The University of Osaka 2-2 Yamadaoka, Suita Osaka 565-0871 Japan; h Global Center for Medical Engineering and Informatics, The University of Osaka 2-2 Yamadaoka, Suita Osaka 565-0871 Japan; i R3 Institute for Newly-Emerging Science Design, The University of Osaka 1-3 Machikaneyamacho Toyonaka Osaka 560-8531 Japan

## Abstract

Microplastics (MPs) are defined as plastic particles smaller than 5 mm, while nanoplastics (NPs) are particles with a diameter less than 1 µm. Both are abundant in the environment and human exposure to them is inevitable. To fully understand the risks posed by plastics to humans, understanding their hazards and kinetics in the body is crucial. However, currently available particles for kinetic analysis, especially for NPs, are limited to spherical polystyrene polymers. This study assessed polyethylene (PE) and prepared PE NPs (nPE) fluorescently labeled with quantum dots (Qdots). Fluorescently labeled nPE was prepared by dissolving and reprecipitating PE MPs (mPE) using Qdots, very small fluorescent particles containing zinc and indium. Microscopic observation and particle size distribution measurements showed that spherical particles smaller than 1 µm were prepared. ATR-IR analysis showed that the fluorescently labeled nPE did not alter surface properties. Fluorescence intensity measurements showed that the fluorescence was sufficiently high for analysis. Evaluation of the fluorescence stability of green and red fluorescent nPE showed that the fluorescence did not fade after one-week suspension in ethanol. Additionally, ICP-MS analysis revealed that Qdots were incorporated into the particles. Cellular uptake assays demonstrated that surface-oxidized labeled nPE exhibited a greater tendency for adhesion to and internalization by a monocyte-derived macrophage-like cell line. This method is versatile and can be adapted to label other polymer types. Collectively, the labeling method established in this study is expected to be useful for analyzing the intracellular and *in vivo* kinetics of NPs in the environment.

## Abstract 2

A platform for Qdots labeled polyethylene nanoplastics is established.

## Graphical abstract

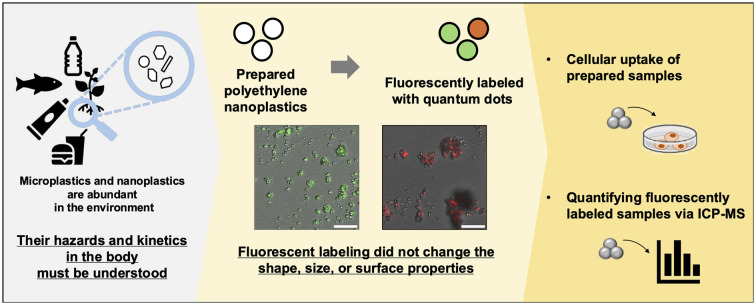

## Introduction

The amount of plastic waste generated has been increasing in recent years.^1^ To mitigate this environmental problems, multiple approaches have been undertaken, such as reducing plastic waste generation, developing technologies for environmental removal, and research focusing on the ecological and biological effects of plastic debris, including their potential impacts on human health.^2–4^ Microplastics (MPs) are plastic particles with diameters less than 5 mm, and concerns regarding them have increased.^5^ Meanwhile, nanoplastics (NPs) have been newly defined as plastic particles with a diameter less than 1 µm, and health concerns are increasing owing to their small size.^1^ They have been detected in the ocean, air, and soil,^6,7^ and humans cannot avoid exposure to MPs and NPs (MNPs) through ingestion, inhalation, or skin contact.^8^ Regarding human-health effects, MNPs have been detected in areas such as the blood, placenta, lungs, and feces.^6,9–11^ Moreover, a relationship exists between MNPs and human diseases such as Inflammatory Bowel Disease and cardiovascular events.^12,13^ Despite the importance of understanding the biological effects of MNPs, relevant knowledge is limited.

Therefore, to fully comprehend the risks of MNPs in humans, understanding their hazards and kinetics in the body is crucial. The dynamics of polystyrene (PS) NPs have been found to vary depending on their size and surface characteristics. A study examining the protein corona of NPs of different sizes and surface charges found that negatively charged COOH-NPs accumulated in the lungs more effectively than positively charged NH_2_-NPs. The study also showed that 100 nm NPs accumulated more in the lungs, compared to larger particles. These results underscore the importance of the size and surface charge of NPs in influencing their distribution in the body.^14^ Another study found that smaller NPs were more readily absorbed by the liver, promoted reactive oxygen species (ROS) generation in zebrafish larvae, stimulated steroid hormone biosynthesis, and influenced the activity of various immune cells, potentially leading to immune-related diseases.^15^ However, currently available particles for kinetic analysis, especially for NPs, are limited to spherical PS polymers. NPs in the environment have complex physicochemical properties (*e.g.*, size, shape, surface characteristics, and polymer types).^16^ Hence, NPs with various physicochemical properties suitable for kinetic analyses are necessary. If these particles are available, they can be used not only for kinetic analyses but also to understand how NPs enter and are distributed in the body.

This focused on polyethylene (PE), which has among the highest production volumes,^17^ and prepared and validated PE NPs (nPE) fluorescently labeled with quantum dots (Qdots).

## Materials and methods

### Reagents

InP/ZnS Qdots were purchased from Sigma-Aldrich (Cat. No. 776750 [fluorescence *λ*_em_: 530 nm] and 776 777 [fluorescence *λ*_em_: 620 nm]; St. Louis, MO, USA). PE particles (Flo-thene), UF20S, with a medium particle size of 24 µm, per the vendor's information, were purchased from Sumitomo Seika Chemicals (Osaka, Japan).

### Sample preparation

nPE was prepared by modifying a previously reported protocol.^18^ The PE particles were dissolved in xylene at 110 °C, reprecipitated *via* dropwise addition into 100 mL of dimethyl sulfoxide (DMSO) with stirring using a magnetic stir bar set at 1000 rpm. Before adding the PE in the xylene to DMSO, 20 µL of 5 mg mL^−1^ Qdots, placed in toluene, was added to the DMSO to label the particles. nPE was collected *via* filtration using a polyester track-etched membrane filter with a 0.2 µm pore size (Cat. No. 1215288; GVS S.p.A., Bologna, Italy). The nPE on the filter was collected *via* sonication with *tert*-butyl alcohol, followed by freeze-drying using a cold-trap device (UT-1010; EYELA, Tokyo, Japan)."

### Particle shape, zeta potential and size distribution analysis

Images of the particles were captured using a JSM-F100 scanning electron microscope (SEM) (JSM-F100, JEOL Ltd., Tokyo, Japan) or S-4800 SEM (HITACHI, Tokyo, Japan), while the particle size distribution was measured using Zetasizer Nano-S (Malvern Panalytical, Worcestershire, UK) or ELSZneoSE (Otsuka Electronics, Osaka, Japan).

### ATR-IR measurement

To assess the surface characteristics of nPE, attenuated total reflection infrared (ATR-IR) spectra were acquired using FT/IR-4X (JASCO, Tokyo, Japan) equipped with a Tin Oxide Gas Sensor (TGS) detector. A diamond ATR crystal, set at an incident angle of 45° to achieve approximately one reflection, was utilized within a horizontal ATR accessory for sample measurement. Spectra were collected over a 7800–400 cm^−1^ range with 32 scans at a resolution of 4 cm^−1^. Initially, a background spectrum without any sample (air) was recorded on the ATR crystal, after which sample measurements were performed. The specimens were then placed on an ATR crystal and pressed to ensure adequate contact. Subsequently, the IR spectra of the PE samples were obtained. The raw spectra were presented as the pATR (=−log *I*/*I*_0_) spectra, where the sample spectral intensity (*I*) was normalized by the background spectral intensity (*I*_0_) just before sample measurement. Each value was normalized to the maximum value for each condition to normalize the IR spectra.

### Particle imaging analysis

Fluorescent-labeled nPE was imaged using CellVoyager CV8000 (Yokogawa, Tokyo, Japan) with 405 nm excitation and 525/50 nm emission filters for green Qdots, and 405 nm excitation and 600/37 nm emission filters for red Qdots. Thereafter, the images were processed and analyzed using the Fiji software (version 2.14.0/1.54i, National Institutes of Health, Bethesda, MD, USA).

### Fluorescence measurement and stability test

The fluorescence values for green and red fluorescently labeled nPE in EtOH (0.0625, 0.125, 0.25, 0.5, and 1 mg mL^−1^) were measured using a plate reader (infinite M1000; TECAN, Männedorf, Switzerland). In addition, green and red fluorescently labeled nPE was dispersed in EtOH and left for 0, 24, and 72 h and for 1 week at room temperature. The nPE and ethanol were then separated *via* centrifugation (10 000×*g*, 5 min), and the fluorescence intensities of the supernatant and green and red nPE particles were measured using a plate reader (infinite M1000, TECAN) and fluorescent images were imaged using a CV8000 (Yokogawa).

### Cytotoxicity assay

The human monocyte cell line THP-1 was obtained from the American Type Culture Collection (Manassas, VA, USA) and were cultured in RPMI-1640 (FUJIFILM Wako Pure Chemical Corporation) supplemented with 10% fetal bovine serum (FBS; Biosera, Nuaille, France), 1% (v/v) penicillin–streptomycin–amphotericin B suspension (Ab; FUJIFILM Wako Pure Chemical Corporation), and 0.1% 2-mercaptoethanol (Thermo Fisher Scientific, Waltham, MA, USA). Cells were maintained at 37 °C in 5% CO_2_ and > 95% humidity. THP-1 cells (2.0 × 10^4^ cells per well) were seeded in 96-well plates and differentiated with 0.5 nM phorbol 12-myristate 13-acetate (PMA; Sigma-Aldrich) for 24 h. The medium was then replaced with 50 µL medium containing nPE in 0.1% carboxymethyl cellulose sodium salt (CMC; FUJIFILM Wako Pure Chemical Corporation). Cells were incubated with continuous shaking at 100 rpm for 24 h. Cell viability was evaluated using the 3-(4,5-dimethyl-thiazol-2-yl)-2,5-diphenyl tetrazolium bromide (MTT; Tokyo Chemical Industry Co., Tokyo, Japan).

### Cell uptake assay

THP-1 cells (2.0 × 10^4^ cells per well) were seeded in 96-well plates and differentiated with 0.5 nM PMA for 24 h. The medium was then replaced with 50 µL medium containing nPE in 0.1% CMC. Cells were incubated with continuous shaking at 100 rpm for 24 h. Cells were imaged using a CV8000 (Yokogawa). Cells with nPE were manually counted.

### Statistical analyses

Statistical analyses were performed using Prism 10.2.3 for MacOS (GraphPad, San Francisco, CA, USA). Data are presented as means ± standard deviation (SD). *P*-values were calculated using one-way analysis of variance (ANOVA), followed by Tukey's test. Statistical significance was set at *P* < 0.05. Graphs were generated using Prism 10.2.3 for MacOS.

## Results and discussion

### SEM images and particle size distribution of prepared particles

**Fig. 1:**
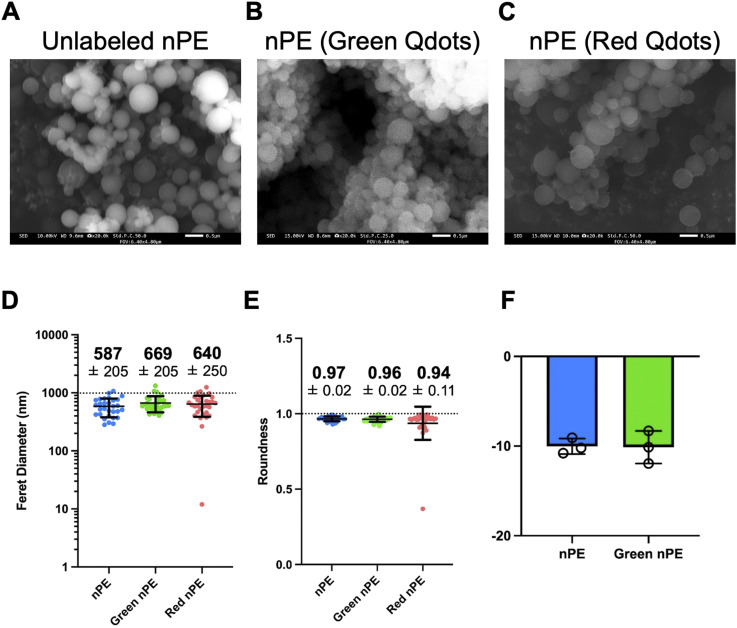
Scanning emission microscopy (SEM) images and particle size distribution and zeta potential of the prepared particles. SEM images of unlabeled polyethylene nanoplastics (nPE) (A), and nPE utilizing Qdots (B and C). Scale bars: 1 µm. SEM images were analysed using ImageJ software. Feret diameter (D) and roundness (E) of plastic particles are shown. (F) Zeta potential of unlabeled polyethylene nanoplastics (nPE), green fluorescent labeled nPE (1 mg mL^−1^) in milliQ containing 0.05% tween are shown.

Using the method of previous study,^18^ with some modification, PE particles were dissolved in xylene and reprecipitated *via* dropwise addition to DMSO with stirring. Before adding the PE particles in the xylene to DMSO, Qdots were added to the DMSO to label the particles. nPE was collected *via* filtration using a filter with a 0.2 µm pore size. nPE on the filter was collected *via* sonication with *tert*-butyl alcohol, followed by freeze-drying using a cold-trap device. To investigate the shape and size of the prepared particles, their morphological features were observed by SEM, and particle size and shape were further analyzed by image analysis of the SEM micrographs. SEM images of the unlabeled particles and nPE utilizing Qdots are shown in Fig. 1. These particles had a spherical shape and a size less than 1 µm (Fig. 1A–C). SEM images were analyzed using ImageJ software to determine the Feret diameter and roundness, which represent particle size and the degree of sphericity, respectively. The Feret diameters of all prepared particles were less than 1 µm, with an average diameter of approximately 600 nm (Fig. 1D). Regarding roundness, all particle types exhibited values around 0.95, indicating nearly perfect spherical shapes (Fig. 1E). These results indicate nPE utilizing Qdots exhibited a surface morphology similar to that of unlabeled nPE, indicating that adding Qdots did not affect the morphological characteristics of the final prepared particles. Due to the tendency of the polymer particles to aggregate, dynamic light scattering (DLS) analysis was performed in an ethanol-based solution to facilitate particle dispersion. The results of the particle size distribution revealed that the average particle size of unlabeled particles was 735.4 nm, which is less than 1 µm (Fig. S1). Based on these results, the NPs were successfully prepared in terms of their morphological features. Zeta potential is also an important parameter for particle characterization. Therefore, to evaluate the effect of labeling on the zeta potential, DLS measurements of unlabeled nPE and Green nPE were performed. The results indicated that there was no clear difference between the two types of nPE (Fig. 1F). These findings suggest that labeling with Qdot did not affect the surface charge of nPE.

### Fluorescence imaging and intensity measurement of fluorescently labeled nPE

**Fig. 2:**
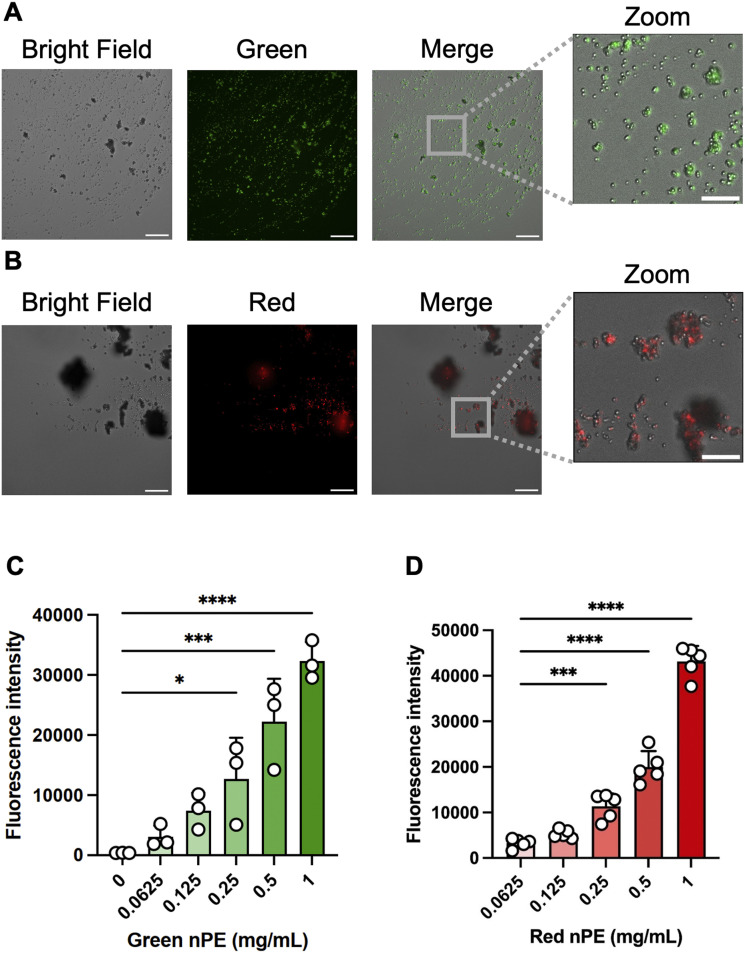
Fluorescence images of fluorescently labeled polyethylene nanoplastics (nPE) and measurement of fluorescence intensity using a plate reader. Fluorescence images of green fluorescently labeled nPE (405 nm excitation and 525/50 nm emission filter) (A) and red fluorescently labeled nPE (405 nm excitation and 600/37 nm emission filter) (B). Scale bars: 30 µm. Scale bars in zoomed images are 10 µm. These images are representative images from three viewpoints. (C) Measurement of fluorescence intensity of 0.0625, 0.125, 0.25, 0.5, and 1 mg mL^−1^ of green (C) and red (D) fluorescently labeled nPE in EtOH, performed using a plate reader. These experiments were repeated twice, with similar results. Significance in (C) and (D) was assessed using one way analysis of variance followed by Dunnett's multiple-comparison test (*, *P* < 0.05; ***, *P* < 0.001; ****, *P* < 0.0001).

**Fig. 3:**
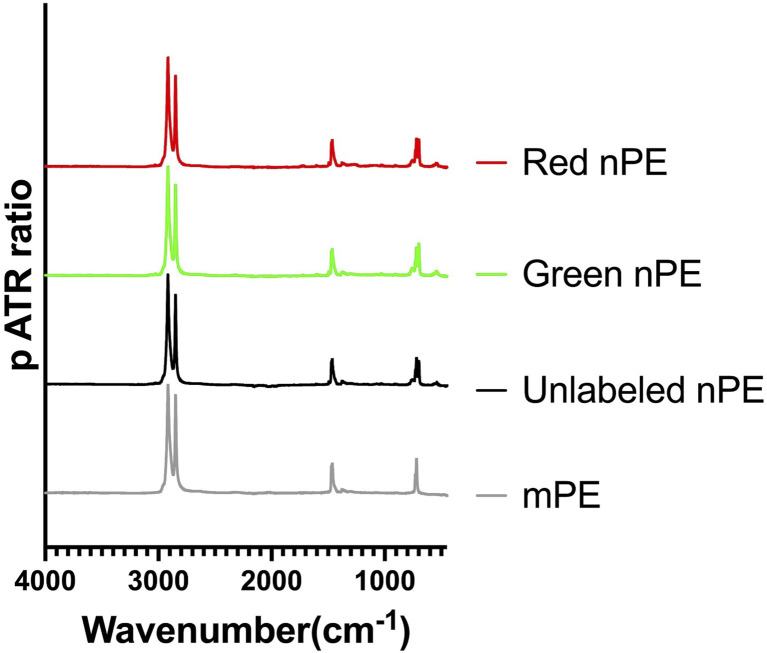
Relative ATR-IR spectra of original PE microplastics (mPE), unlabeled polyethylene nanoplastics (nPE), green fluorescently labeled nPE, and red fluorescently labeled nPE.

Fluorescence microscopic analysis was conducted to confirm that the labeling of nPE with Qdots was successful. Fluorescence images of green fluorescent-labeled nPE (Fig. 2A) and red fluorescent-labeled nPE (Fig. 2B) were captured at 405 nm excitation. Green fluorescence was detected using a 525/50 nm emission filter, while red fluorescence was detected using a 600/37 nm emission filter. As shown in the fluorescence images, the nPE particles were properly labeled with Qdots, indicating that the fluorescence-labeled samples were successfully prepared. To confirm the fluorescence intensity of the fluorescent-labeled nPE, a plate reader was used to measure the fluorescent signal from the particles. At concentrations of 0.0625, 0.125, 0.25, 0.5, and 1 mg mL^−1^, green and red fluorescently labeled nPE were dissolved in ethanol in a 96-well plate, and the fluorescence intensity was measured using a plate reader. A concentration-dependent increase in fluorescence intensity in both green and red fluorescently labeled nPE was observed (Fig. 2C and D). The results suggest that the Qdot inclusion rate in the particles was sufficiently high for analysis, making these particles suitable for kinetic analysis and tracking localization. ATR-IR analysis was conducted to confirm that the fluorescent labeling did not alter the surface characteristics, particularly the functional groups. The ATR-IR spectra of the original PE MPs (mPE), unlabeled nPE, and green and red fluorescence-labeled nPE are shown in Fig. 3. All the samples exhibited C–H stretching bands at around 2900 cm^−1^ and C–H bending bands around 1500 cm^−1^ and 700 cm^−1^, both characteristic of PE polymers. The green- and red-labeled nPE with Qdots showed no changes in surface characteristics compared to those of the original mPE, suggesting that the Qdots might have not adhered to the plastic surface but were internalized. Based on the above findings, the nPE particles were successfully labeled with Qdots and exhibited effective fluorescence without changing surface properties. By encapsulating Qdots, the fluorescence wavelength and intensity theoretically remain unchanged regardless of external polarity. Additionally, unlike fluorescent organic dyes, which tend to adhere to the surface and potentially alter the plastic properties, our method effectively overcomes this limitation. Although it is challenging to determine the exact number of Qdot per particle, the concentration-dependent increase in fluorescence intensity indicates that the fluorescence labeling is uniformly distributed across the particles (Fig. 2C). Recent studies have demonstrated that the fluorescence properties of inorganic nanomaterials can be tuned through structural design and specific interactions with target analytes, and that selective binding or exchange processes can lead to strong fluorescence responses or quenching at low concentrations.^19,20^ These examples illustrate that fluorescence changes in labeled nanoparticles, including Qdot-labeled nanoplastics, may arise not only from particle presence but also from specific environmental or surface interactions that modulate emission intensity.

### Evaluation of fluorescence stability of fluorescently labeled nPE

**Fig. 4:**
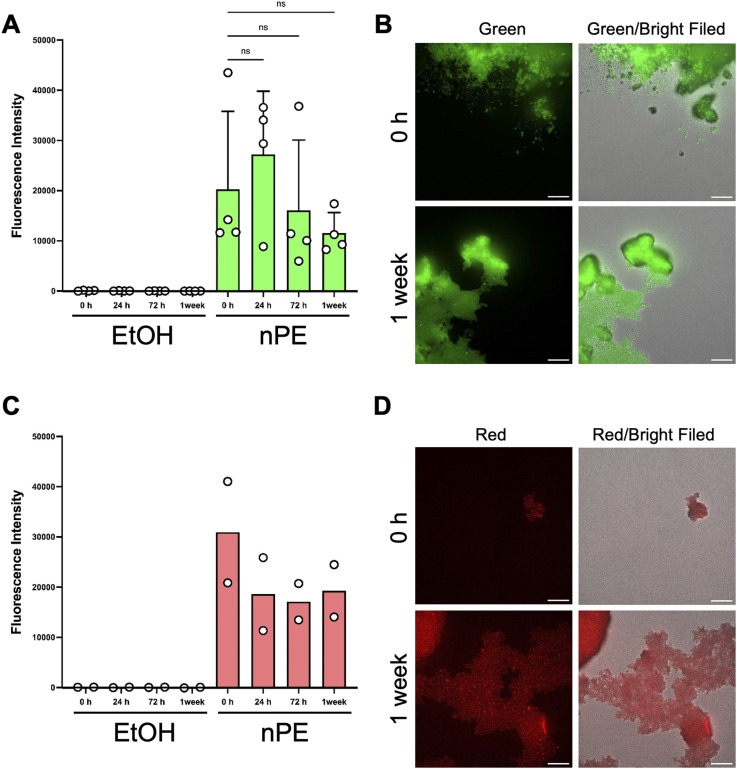
Evaluation of fluorescence stability of green fluorescently labeled nPE. (A) Green fluorescently labeled nPE was dispersed in EtOH, and the fluorescence intensity of supernatant or particles was measured at 0, 24, and 72 h and at 1 week, using a plate reader. The data shows mean ± standard deviation from four independent experiments. (B) Fluorescence images of green fluorescently labeled nPE at 0 h and 1 week after incubation in EtOH (405 nm excitation and 525/50 nm emission filter). Scale bars: 30 µm. Representative images from four experiments are displayed. (C) Red fluorescently labeled nPE was dispersed in EtOH, and the fluorescence intensity of supernatant or particles was measured at 0, 24, and 72 h and at 1 week, using a plate reader. The data shows mean from two independent experiments. (D) Fluorescence images of red fluorescently labeled nPE at 0 h and 1 week after incubation in EtOH (405 nm excitation and 600/37 nm emission filter). Scale bars: 30 µm. Representative images from two experiments are displayed. Significance was assessed using one way analysis of variance, followed by Tukey's method (n.s., not significant).

To utilize fluorescently labeled nPE *in vivo* or *in vitro*, these particles must have sufficient fluorescence stability under long-term experimental conditions. Therefore, the fluorescence stability of green fluorescent-labeled nPE and red fluorescent-labeled nPE in ethanol was tested due to its high dispersibility in ethanol. Fluorescence-labeled nPE was dispersed in ethanol, and the fluorescence intensity was measured using a plate reader at 0, 24, and 72 h and at one week. The fluorescence intensity did not change after 24 and 72 h and after one week in both green fluorescent-labeled nPE and red fluorescent-labeled nPE (Fig. 4A–D). Using fluorescence microscopy, the nPE after one week of dispersion in ethanol was compared with the original fluorescent-labeled nPE. Although a certain degree of aggregation was observed, which is inherent to the physicochemical properties of plastic particles, fluorescence microscopy revealed that the nPE after one week of dispersion in ethanol showed no fluorescence fading compared with the original fluorescent-labeled nPE (Fig. 4B and D). Although further evaluation of its stability in biological fluids is required to determine its suitability for animal administration, the ability to prepare fluorescently labeled nPE with high fluorescence stability suggests the possibility of future applications in dynamic analysis.

Using fluorescently labeled NPs, preferably those emitting at longer wavelengths, *in vivo* imaging studies could be applied for kinetic analyses. Furthermore, fluorescently labeled nPE is expected to be applicable to the selective detection of nPE in complex matrices, including tissue homogenates and soil or sediment samples. Additionally, Qdot-labeled NPs could be used for intracellular localization studies *via* real-time cellular imaging. Fluorescently labeled plastics have been used for dynamic analyses in mice.^21,22^ Unlike conventional fluorescent dyes, such as Rhodamine B, Fluorescein, and Xylene cyanol, Qdot possess unique properties owing to their zinc and indium-based core, which can enable quantitative measurements through inductively coupled plasma-mass spectrometry (ICP-MS). Their distinctive composition allows for quantitative kinetic analysis evaluation in addition to qualitative evaluation, compared to traditional dyes. To evaluate the amount of Qdots incorporated into nPE, we performed ICP-MS analysis to quantify metal elements, such as indium, in Qdot-encapsulated nPE. As a result, indium was detected in the labeled nPE (Fig. S2). In a single preparation, approximately 34 mg of plastic could be prepared, and 80 µL of Qdot solution (5 mg mL^−1^) was added, corresponding to a total Qdot amount of 0.4 mg. Therefore, the theoretical Qdot content per 1 mg of nPE is calculated to be approximately 0.01 mg. However, when the Qdot content was experimentally quantified using the calibration curve constructed with Qdot and count per second (CPS) of Indium, it was found that 1 mg of nPE contained approximately 0.001 mg of Qdot. This indicates that about 10% of the initially added Qdot was incorporated into nPE. Although further improvement of the encapsulation efficiency, for example by modifying the surface chemistry prior to mixing Qdots with the polymer solution, should be addressed in future studies, we confirmed that our labeled nPE successfully encapsulated Qdots. Collectively, this approach highlights the versatility of Qdots not only as imaging agents but also as valuable tools for precise quantification in tissue and cellular environments.^23^

### Evaluation of cellular response of fluorescently labeled nPE

**Fig. 5:**
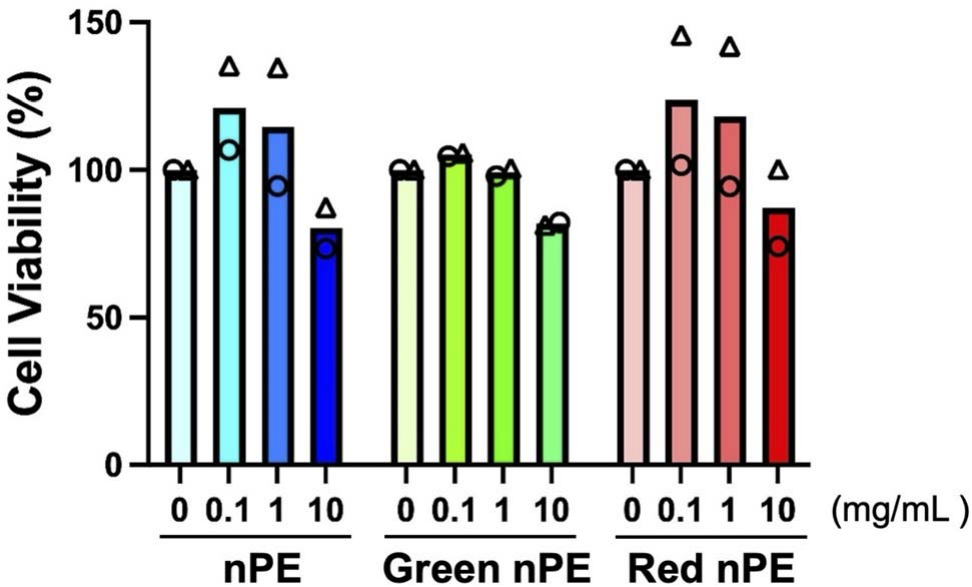
THP-1 cells (2.0 × 104 cells per well) were seeded in 96 well plates and differentiated with PMA for 24 h. Cells were then exposed to unlabeled nPE, green fluorescent labeled nPE and red fluorescent labeled nPE (0, 0.1, 1.0, or 10 mg mL^−1^ in 0.1% CMC medium) under continuous shaking for 24 h, followed by MTT assay. Data represent from two independent experiments.

To investigate cellular responses to fluorescently labeled nPE, cytotoxicity was evaluated using an MTT assay. The THP-1 cell line was selected because MNPs have been detected in multiple organs,^6^ suggesting potential interactions with tissue-resident macrophages.^24^ In addition, macrophages possess a high phagocytic capacity.^25^ PMA-differentiated THP-1 cells are commonly used as a macrophage model due to their macrophage-like characteristics. After exposure of THP-1 cells to nPE, green fluorescent-labeled nPE, or red fluorescent-labeled nPE at concentrations of 0.1, 1, and 10 mg mL^−1^, no marked cytotoxicity was observed (Fig. 5). These results indicate that the prepared fluorescently labeled nPE exhibited no detectable cytotoxic effects and can be utilized for *in vivo* dynamics analysis.

### Application of fluorescently labeled nPE *in vitro*

**Fig. 6:**
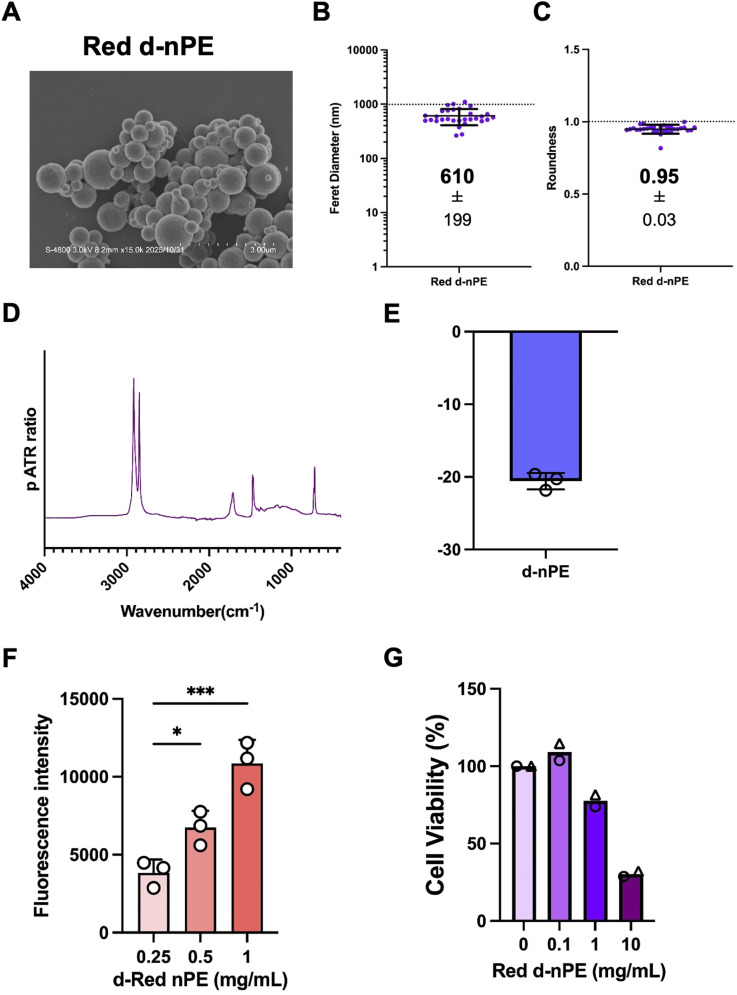
Physicochemical properties of degraded labelled nPE. (A) SEM images of red fluorescent labeled degraded nPE (Red d-nPE). Scale bars: 3 µm. Feret diameter (B) and roundness (C) of red d-nPE particles calculated through SEM image analysis. (D) Relative ATR-IR spectra of Red d-nPE. (E) Zeta potential of Red d-nPE (1 mg mL^−1^) in milliQ containing 0.05% tween. (F) Measurement of fluorescence intensity of 0.25, 0.5, 1 mg mL^−1^ red d-nPE in EtOH using a plate reader. These experiments were repeated twice with similar results. Note that significance was assessed in using one way ANOVA followed by Dunnett's multiple comparison test as follows: *, *P* < 0.05; ***, *P* < 0.001. (G) THP-1 cells (2.0 × 10^4^ cells per well) were seeded in 96 well plates and differentiated with PMA for 24 h. Cells were then exposed to red d-nPE (0, 0.1, 1.0, or 10 mg mL^−1^ in 0.1% CMC medium) under continuous shaking for 24 h, followed by MTT assay. Data represent from two independent experiments.

To evaluate the usability of the prepared fluorescently labeled nPE in biological assays, a cellular uptake assay was conducted. In this experiment, red fluorescently labeled nPE was used because its longer excitation wavelength is advantageous for biological applications. Prior to the cellular uptake assay, environmental relevance was considered. In environmental MNPs, surface oxidation occurs as a result of UV exposure.^26^ To mimic this process, we previously established a method to induce surface oxidation using vacuum ultraviolet (VUV) irradiation at a wavelength of 172 nm.^27–31^ Using this approach, fluorescently labeled nPE was subjected to surface oxidation. After oxidation, SEM observation and subsequent image analysis demonstrated that the surface-oxidized fluorescently labeled nPE retained an average particle size of approximately 600 nm and a spherical morphology (Fig. 6A–C). ATR-IR analysis revealed that, in addition to the peaks present in the original nPE, peaks corresponding to carbonyl groups were increased, indicating successful surface oxidation (Fig. 6D). Furthermore, DLS analysis confirmed the acquisition of a negative surface charge (Fig. 6E). Importantly, this method introduced surface oxidation without altering the original particle size and shape, enabling assessment of the specific effects of surface oxidation.

With respect to fluorescence intensity, VUV exposure resulted in a decrease in fluorescence; however, a concentration-dependent increase in signal intensity was still observed (Fig. 6F). As an initial evaluation of the cellular response to surface-oxidized fluorescently labeled nPE, cytotoxicity was assessed using THP-1 cells. The results showed concentration-dependent cytotoxicity after 24 h of particle exposure, which was comparable to our previous study (Fig. 6G). Using red fluorescently labeled nPE, surface-oxidized fluorescently labeled nPE was exposed to THP-1 cells at 1 mg mL^−1^ for 24 h, and cellular uptake was visualized using high-throughput confocal microscopy.

It has been reported that surface-oxidized polymer particles exhibit autofluorescence.^32^ Oxidation introduces oxygen-containing functional groups, such as carbonyl groups, onto the polymer surface. When these groups are locally clustered, they can generate intrinsic fluorescence through through–space interactions, a phenomenon known as clustering-triggered emission (CTE). Thus, oxidation-induced autofluorescence originates from the polymer particles themselves.^32^

**Fig. 7:**
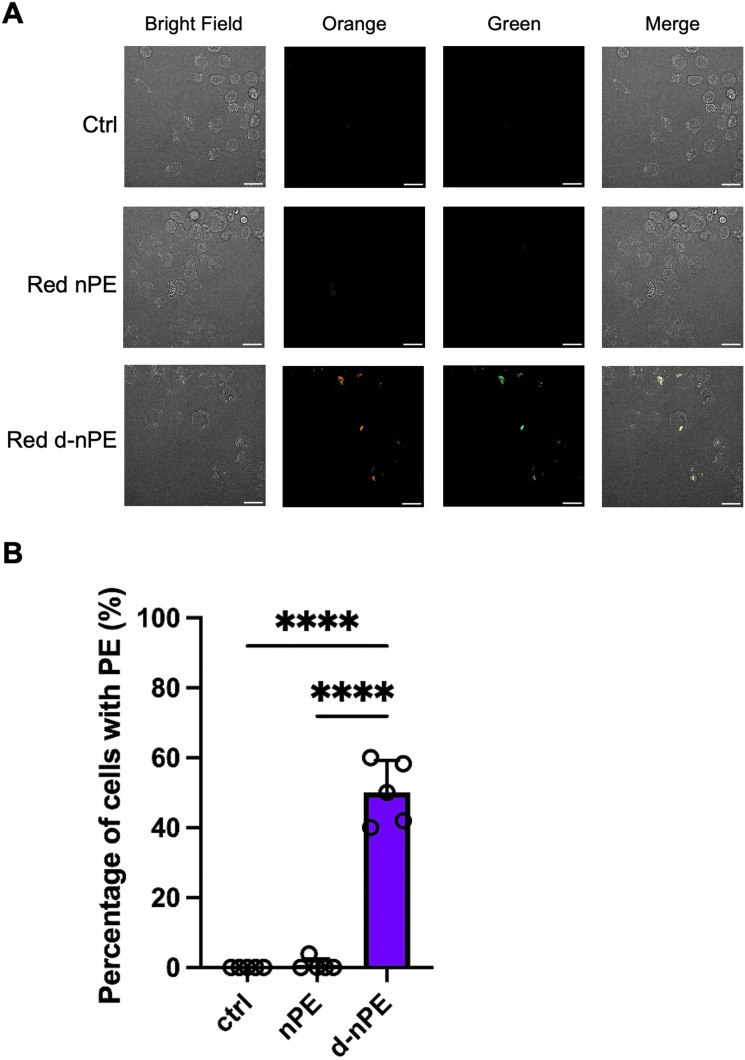
Cellular uptake of labelled nPE. (A) THP-1 cells (2.0 × 10^4^ cells per well) were seeded in 96 well plates and differentiated with PMA for 24 h. Cells were then exposed to red fluorescent labeled nPE and red fluorescent labeled d-nPE (1.0 mg mL^−1^ in 0.1% CMC medium) under continuous shaking for 24 h at 37 °C. (B) The proportion of cells with adherent/incorporated nPE and d-nPE was calculated from the obtained fluorescence images. These experiments were repeated twice with similar results. Note that significance was assessed in using one way ANOVA followed by Tukey's test as follows: ****, *P* < 0.0001.

Accordingly, after incubation with labeled nPE, non-oxidized fluorescently labeled nPE was detected using 405 nm excitation and a 600/37 nm emission filter for red fluorescence. In addition to this channel, autofluorescence from oxidized fluorescently labeled nPE was detected using 405 nm excitation and a 525/50 nm emission filter. As a result, few cells were positive for non-oxidized nPE, whereas oxidized nPE was taken up by a greater number of cells (Fig. 7A). Particles attached to or internalized by cells were quantified, and the results showed significantly higher cell–particle interactions for oxidized particles than for pristine nPE, indicating enhanced cellular uptake of oxidized nPE (Fig. 7B). These findings demonstrate that the prepared fluorescently labeled nPE can be applied to cellular uptake assays. In addition, although longer incubation periods (*e.g.*, 1 week) should be evaluated in future studies, the present results indicate that the fluorescence signal is sufficiently stable under biological conditions.

From a sustainability perspective, the reusability of nPE would be desirable; however, in the present study, the labeled nPE could not be reused after biological exposure, which should be considered a limitation.

## Conclusions

In this study, we developed a method to prepare fluorescently labeled nPE using Qdots. The resulting nPE, smaller than 1 µm, showed stable fluorescence and maintained surface properties, making them suitable for analysis. Kinetic analyses using PS have been conducted in various models, such as zebrafish and mice;^22,33,34^ however, studies involving other polymer types remain limited. The method in this study can be adapted to label other polymer types, such as polypropylene and polyvinyl chloride, facilitating broader comparisons. Comparative studies of different polymer types can help elucidate their distinct biological impacts. Furthermore, environmental plastics not only vary in polymer type but also in shape and surface properties. We have shown that degraded samples that mimic plastics in the environment have stronger cytotoxicity than that of undegraded samples.^28,29^ By applying the methodology used in this study, future research can explore the precise control of the shape and surface properties of NPs, allowing for more accurate representations of environmental samples and a deeper understanding of their behavior within the human body. NPs can enter the body *via* various exposure routes, including oral ingestion, inhalation, and dermal absorption.^35^ To effectively assess the impact of NPs on human health, it is crucial to conduct *in vivo* studies that consider these diverse routes of exposure. Such studies would provide a more comprehensive understanding of the potential risks associated with NPs exposure and help inform regulatory policies aimed at mitigating these risks. This labeling technique, adaptable for other polymers, holds potential for quantitative measurements and studying the behavior of nanoparticles in intracellular and *in vivo* environments.

## Author contributions

Mii Hokaku: data curation, investigation, validation, visualization, formal analysis, writing – review & editing and writing – original draft; Yuya Haga: investigation, visualization, formal analysis, writing – original draft, writing – review & editing, conceptualization, project administration, and methodology; Kosuke Tanaka: conceptualization, methodology, and writing – review & editing; Hirofumi Tsujino: conceptualization, and writing – review & editing; Haruyasu Asahara: writing – review & editing; Kazuma Higashisaka: writing – review & editing; Yasuo Tsutsumi: writing – review & editing, supervision, funding acquisition, project administration, and conceptualization.

## Conflicts of interest

There are no conflicts of interest to declare.

## Supplementary Material

RA-016-D6RA01781A-s001

## Data Availability

All relevant data are provided in this article. The data supporting the findings of this study are available from the corresponding author upon reasonable request. Supplementary information (SI) is available. See DOI: https://doi.org/10.1039/d6ra01781a.
