# Supplementary material for: Development of a platform for quantum-dot-labeled polyethylene nanoplastics for dynamic analytical applications
Source: RSC Adv. 2026 Apr 7;16(20):18199–209. doi: 10.1039/d6ra01781a (PMC13054805; doi:10.1039/d6ra01781a)
Supplement: RA-016-D6RA01781A-s001 [file RA-016-D6RA01781A-s001.pdf]

Supplementary Information (SI) for RSC Advances.
This journal is © The Royal Society of Chemistry 2026

ICP-MS measurement

nPE was treated with mixed acid (60% nitric acid, 30% hydrogen peroxide and ultrapure water mixed at a ratio of 8:3:9; Wako, Osaka, Japan), and shaken at 99° C for 1 h to melt the plastic particles. Samples were diluted 100 times with ultrapure water. The total indium concentration in the samples was measured by inductively coupled plasma mass spectrometry (ICP-MS) using an Agilent 7800 instrument (Agilent Technologies; Santa Clara, CA, USA). The analysis conditions were as follows: RF power, 1550 W; carrier gas, 1.01 L/min Ar; and dwell time, 0.1 s. Rhodium and thallium were used as the internal standard for indium. The target elements were 103Rh, 115In, 118In and 205Tl. Calibration curve was generated using 115In standard solution. Metal standard solutions were obtained from FUJIFILM Wako Pure Chemical Corporation (Osaka, Japan).

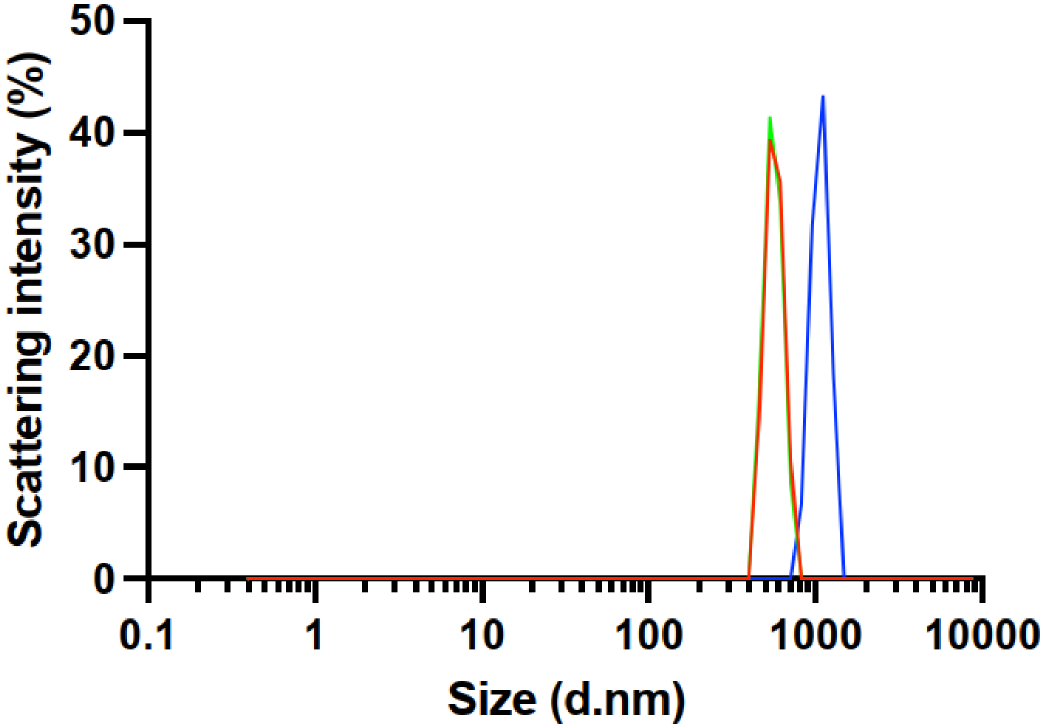

Supplementary figure 1. Volume-based frequency distribution of unlabeled nPE (1 mg/mL in ethanol). Data are expressed as triplicates. This experiment was repeated, with similar results.

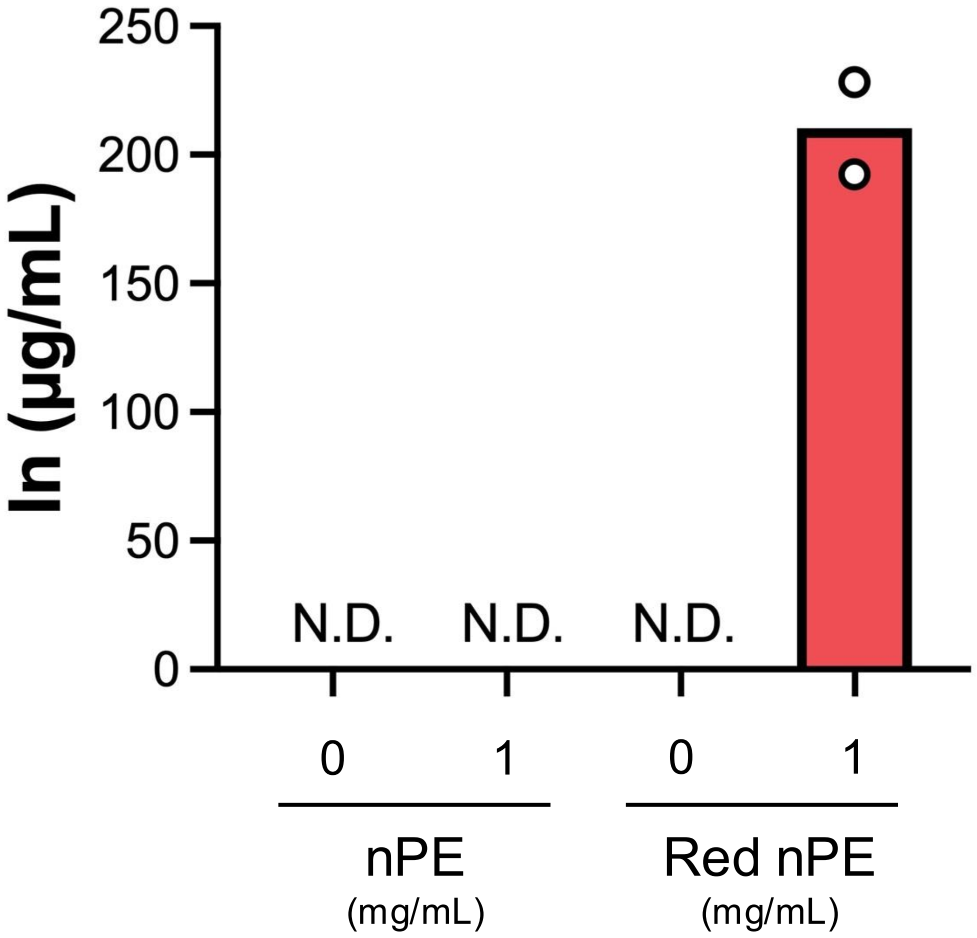

Supplementary figure 2. Unlabeled nPE and fluorescent-labeled nPE were analysed by ICP-MS. nPE was diluted with 0.05% tween in ultra-pure water to 1 mg/mL. The indium (In) content in the red fluorescently labeled nPE was quantified using calibration curve of Indium and Rh and Tl as internal standards. For each experiment, the value was calculated using the calibration curve obtained in that run and the mean counts per second (CPS) value from three technical replicates. Each calculated value is shown as one dot. Results from two independent experiments are presented, with the mean value and the individual dots shown.
